# Linking atrial fibrillation to cerebral small vessel disease: a cross-sectional study with predictive analytics

**DOI:** 10.3389/fcvm.2026.1698488

**Published:** 2026-05-20

**Authors:** Jingzhou Shang, Lu Fan, Jing Xu, Xiaofei Sui

**Affiliations:** 1 Department of Cardiology, Wuhan Hospital of Traditional Chinese Medicine, Wuhan, Hubei, China; 2 Department of Neurology, Renmin Hospital, Hubei University of Medicine, Shiyan, Hubei, China

**Keywords:** atrial fibrillation, cerebral microbleeds, cerebral small vessel disease, sex differences, silent brain infarcts, white matter hyperintensities

## Abstract

**Background:**

Atrial fibrillation (AF) and cerebral small vessel disease (CSVD) are prevalent in older adults and may synergistically contribute to cognitive decline and stroke risk. This study investigates the association between AF and CSVD markers, including white matter hyperintensities (WMHs), cerebral microbleeds (CMBs), lacunes, and silent brain infarcts, focusing on sex-specific patterns, anticoagulation status, and predictive modeling.

**Methods:**

In a cross-sectional study of 923 individuals aged ≥70 years undergoing 3.0-Tesla brain MRI, AF was identified via ECG, self-reports, and hospital records. CSVD markers were quantified using standardized MRI protocols. Regression models, adjusted for demographic and vascular risk factors, assessed associations between AF and CSVD markers, stratified by sex, anticoagulation status, and AF onset age. Random Forest and logistic regression models predicted high WMH burden and stroke risk.

**Results:**

Of 923 participants, 85 (9.2%) had AF. AF was associated with higher WMH volumes (adjusted mean: 0.0046 vs. 0.0034 mL/TIV, *p* = 0.051; approaching statistical significance), particularly in men (*p* = 0.072) and in participants receiving anticoagulation therapy (*p* = 0.007). AF increased odds of symptomatic stroke (OR: 4.2, 95% CI: 2.0–8.8), large infarcts, lacunes, and silent infarcts, with stronger effects in men. Frontal lobe CMBs were more prevalent in men with AF (OR: 3.9, *p* = 0.049). Predictive models showed high accuracy for WMH burden (81.2%, AUC-ROC: 0.88) and stroke (84.9%, AUC-ROC: 0.91).

**Conclusion:**

AF is independently associated with CSVD, particularly in men and anticoagulated individuals, emphasizing the need for targeted strategies to mitigate AF-related brain injury.

## 1 Introduction

Cerebral small vessel disease (CSVD) is a prevalent condition in older adults, characterized by neuroimaging findings such as white matter hyperintensities (WMHs), cerebral microbleeds (CMBs), lacunes, and silent brain infarcts, which are linked to cognitive decline, stroke, and increased mortality (1). Atrial fibrillation (AF), one of the most common cardiac arrhythmias, significantly increases the risk of ischemic stroke due to cardioembolic mechanisms (2). Beyond clinical stroke, AF may contribute to CSVD through microembolization, cerebral hypoperfusion, and systemic inflammation, potentially exacerbating subclinical brain injury (3, 4). The interplay between AF and CSVD is of growing interest, as both conditions are highly prevalent in aging populations and may synergistically impair cognitive and functional outcomes (5, 6).

Previous studies have reported associations between AF and increased WMH burden, suggesting that chronic arrhythmia may promote white matter damage through vascular or inflammatory pathways (7, 8). Silent brain infarcts, another hallmark of CSVD, are also more prevalent in individuals with AF, even in the absence of clinical stroke (9, 10). However, the relationship between AF and CMBs remains controversial, with some studies indicating an association, particularly in lobar regions, while others report no significant link (11, 12). These discrepancies may reflect differences in study populations, imaging protocols, or adjustment for confounders such as hypertension and anticoagulant use (13, 14). Sex differences in the cerebral impact of AF are increasingly recognized but remain underexplored. Women with AF may have a higher stroke risk than men, potentially due to hormonal or vascular differences, yet men may exhibit a greater burden of CSVD markers such as WMHs (15, 16). Anticoagulation therapy, a mainstay of AF management for stroke prevention, may further modulate CSVD pathology. For instance, non-vitamin K antagonist oral anticoagulants (NOACs) and warfarin have been associated with varying risks of CMBs, though their impact on WMH progression is less clear (17, 18). Additionally, the age of AF onset may influence the extent of cerebral damage, with early-onset AF potentially leading to a cumulative burden of microvascular injury over time (19, 20).

**Figure 1:**
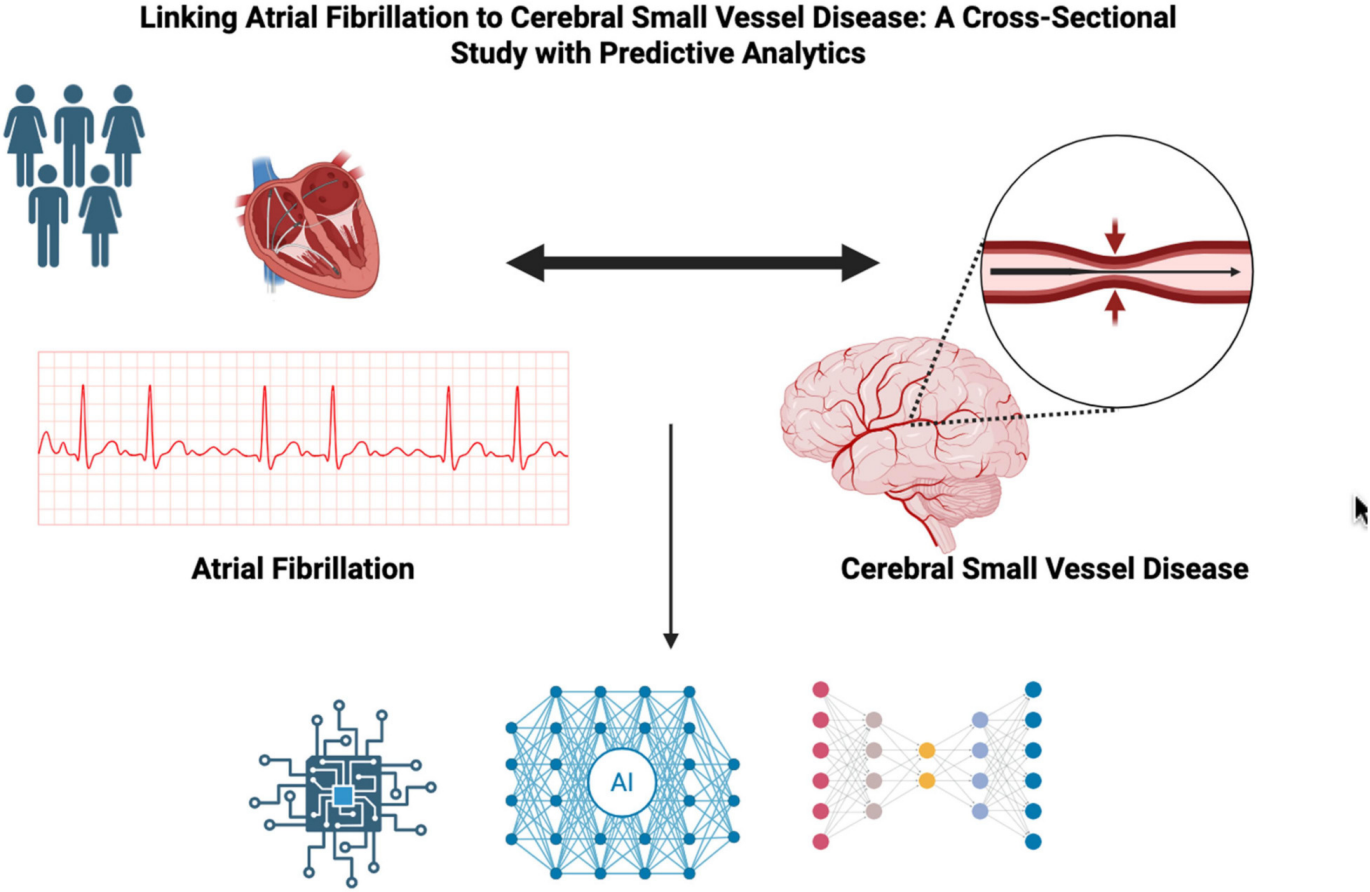
Conceptual overview of the study linking atrial fibrillation to cerebral small vessel disease using predictive analytics. This schematic illustrates the hypothesized relationship between atrial fibrillation (AF) and cerebral small vessel disease (CSVD). AF-related hemodynamic irregularities and embolic mechanisms may contribute to microvascular injury in the brain, manifesting as neuroimaging markers of CSVD. Clinical, demographic, and cardiovascular variables were integrated into machine-learning models, including Random Forest–based predictive analytics, to evaluate associations and identify key predictors of CSVD burden.

The mechanisms linking AF to CSVD are complex and likely involve shared vascular risk factors, such as hypertension and diabetes, as well as AF-specific effects like irregular cerebral blood flow (21, 22). Population-based studies with comprehensive neuroimaging and clinical data are needed to clarify these associations and their implications for brain health. This study investigates the relationship between AF and CSVD markers in a cohort of older adults, with a focus on WMHs, CMBs, and infarcts. By examining sex-specific patterns, anticoagulation status, and age of AF onset, we aim to provide insights into the mechanisms of AF-related brain injury and its contribution to cognitive and vascular outcomes. This study aimed to comprehensively evaluate the relationship between atrial fibrillation (AF) and cerebral small vessel disease (CSVD) by quantifying the prevalence and severity of key neuroimaging markers including white matter hyperintensities (WMHs), cerebral microbleeds (CMBs), lacunes, and silent brain infarcts in individuals with and without AF. A primary objective was to examine the association between AF and WMH volumes using regression models adjusted for demographic, vascular, and clinical confounders, including history of stroke and infarcts. Additionally, we assessed the relationship between AF and the presence of CMBs, both globally and across anatomical subregions (lobar, deep, and specific lobar locations), with appropriate adjustment for relevant risk factors. The study further aimed to evaluate the impact of AF on both clinical and silent infarcts, including large infarcts and lacunes, and to explore potential sex-specific differences in these associations. Given the clinical importance of treatment timing and intensity, we also investigated the modifying effects of anticoagulation therapy and age of AF onset on the relationship between AF and CSVD markers. Lastly, we sought to explore sex-stratified patterns in these associations to identify potential differences in cerebral vulnerability between men and women, thereby contributing to a more individualized understanding of AF-related brain pathology. The Overall flow of the work showed in Figure 1.

## 2 Methods and materials

### 2.1 Study design and participants

This cross-sectional analysis was derived from a population-based study of individuals aged 70 years. A total of 1,358 eligible individuals were invited from the general population registry; of these, 923 (68%) participated in brain MRI and were included in the imaging analysis. Participants signed the written informed consent form, and this research has been approved by the ethics review committee of our hospital. Exclusion criteria included diagnoses of hydrocephalus, multiple sclerosis, Parkinson's disease, congenital brain malformations, or brain tumors. Individuals with incomplete MRI sequences or missing core clinical variables were also excluded.

### 2.2 Atrial fibrillation and clinical evaluation

Atrial fibrillation (AF) status was determined using three sources: 12-lead ECG (coded with Minnesota Code 8–3), self-reported physician-diagnosed AF, and linkage with hospital records using ICD-10 code I48.x. Participants were classified as having AF if any of these sources indicated AF presence. The age at AF onset (<75 vs. ≥75 years) was collected via interviews and clinical records. Anticoagulant use, including warfarin or non–vitamin K antagonist oral anticoagulants (NOACs), was documented based on medication reviews and verified prescriptions. The CHA_2_DS_2_-VASc score was calculated for stroke risk stratification. Demographic data and cardiovascular risk factors (hypertension, diabetes, hypercholesterolemia, BMI, smoking, alcohol intake, and APOE *ε*4 status) were assessed during standardized clinical visits and fasting blood tests.

### 2.3 MRI acquisition and processing

MRI examinations were performed using a 3.0-Tesla scanner with standardized protocols. Sequences included:

- 3D T1-weighted MPRAGE
- T2-weighted FLAIR
- T2*-weighted gradient echo (GRE) for detection of cerebral microbleeds

### 2.4 White matter hyperintensities (WMHs)

WMHs were segmented using the Lesion Segmentation Toolbox (LST) in SPM12 and normalized to total intracranial volume (TIV). Volumes were reported in mL and log-transformed to correct for skewness in statistical analysis. Lesion maps were visually inspected for quality control.

### 2.5 Cerebral microbleeds (CMBs)

CMBs were identified on GRE sequences and rated using the Microbleed Anatomical Rating Scale (MARS). Locations were categorized as lobar (frontal, parietal, temporal, occipital), deep, or any CMBs. Two trained raters blinded to clinical data reviewed all scans; disagreements were resolved by consensus.

### 2.6 Infarcts and lacunes

Lacunes and infarcts were defined per STRIVE criteria and identified by a board-certified neuroradiologist. Silent brain infarcts were included if no clinical stroke was documented but radiographic evidence was present. Infarcts were further categorized into large infarcts and lacunar infarcts.

### 2.7 Cognitive and functional assessment

Global cognitive function was assessed using the Mini-Mental State Examination (MMSE). Dementia was diagnosed based on clinical examination and validated criteria. Stroke history was determined from self-report and verified by medical records.

### 2.8 Statistical analysis

Demographic and clinical characteristics were compared using chi-square or t-tests as appropriate. Multiple linear regression models were used to examine the association between AF and WMH volumes (dependent variable: mL/TIV), and logistic regression was used for associations between AF and CMB presence. Statistical significance was defined as a two-tailed *p*-value < 0.05 unless otherwise specified. No formal adjustment for multiple comparisons was applied, given the exploratory nature of the study and the focus on a limited, pre-specified set of primary CSVD markers (white matter hyperintensity volume, presence of cerebral microbleeds, lacunes, and infarcts) with hypothesis-driven stratification by sex and anticoagulation status. Therefore, findings particularly from stratified and subgroup analyses should be interpreted with caution and regarded as hypothesis-generating.

Four hierarchical models were applied:

- **Model 1:** Unadjusted
- **Model 2:** Adjusted for sex, education, APOE *ε*4, BMI, alcohol use, smoking, hypertension, diabetes, hypercholesterolemia, and heart disease
- **Model 3:** Model 2 + adjustment for symptomatic stroke
- **Model 4:** Model 2 + adjustment for MRI-confirmed infarcts

All WMH values were log-transformed due to non-normal distribution. Analyses were stratified by sex were indicated due to expected biological differences in cerebral small vessel disease. Statistical significance was set at *p* < 0.05. All analyses were conducted using SPSS version 27.0 (IBM Corp.) and R version 4.2.0.

Although multiple subgroup and sex-stratified analyses were performed, these were prespecified and hypothesis-driven, based on prior evidence suggesting sex-related differences in atrial fibrillation–associated cerebrovascular risk and potential modifying effects of anticoagulation status and age at AF onset. The primary analyses were limited to a defined set of cores CSVD markers (white matter hyperintensity volume, cerebral microbleeds, lacunes, and infarcts). Given the focused and biologically motivated nature of these analyses, formal correction for multiple comparisons was not applied. However, findings from subgroup and stratified analyses particularly those with borderline statistical significance should be interpreted cautiously and considered exploratory.

### 2.9 Predictive modeling of white matter hyperintensity burden and stroke

To evaluate the predictive utility of clinical and demographic features in determining cerebral small vessel disease (CSVD) outcomes (Table 1), we developed two supervised learning models using the cross-sectional dataset from our cohort study.

**Table 1:** Demographic, clinical, lifestyle, and genetic variables included in the study cohort, along with their corresponding data types.

Feature	Type
Age	Continuous
Sex	Binary (M/F)
Hypertension	Binary
Diabetes	Binary
Hypercholesterolemia	Binary
Body Mass Index (BMI)	Continuous
Smoking status	Binary
Alcohol risk use (>98 g/w)	Binary
Atrial fibrillation (AF)	Binary
Anticoagulant use	Binary
APOE ε4 genotype	Binary

#### 2.9.1 Dataset and feature selection

The input dataset consisted of 923 individuals aged ≥70 years who underwent 3.0 T brain MRI and clinical evaluation. The dependent variables (prediction targets) were:

- **Model A:** Binary classification of high WMH volume, defined as total WMH volume normalized to total intracranial volume (WMH/TIV) greater than the sample median.
- **Model B:** Binary classification of presence of symptomatic stroke, as determined by clinical history and radiographic verification.

Independent variables (features) used in both models included:

Missing data were imputed using mean values for continuous variables and mode for categorical variables.

#### 2.9.2 Handling of missing data

Participants with missing atrial fibrillation status or incomplete MRI data for primary CSVD outcomes were excluded from the main analyses. For covariates with low levels of missingness (<X%), complete-case analysis was performed. Where appropriate, sensitivity analyses were conducted using multiple imputation under the assumption of missing at random to assess the robustness of findings. Results were materially unchanged, supporting the stability of the reported associations.

#### 2.9.3 Model a: random forest for WMH burden

We trained a Random Forest classifier to model the probability of high WMH burden. Random Forest is an ensemble learning method that aggregates multiple decision trees trained on bootstrapped subsets of the data to improve prediction accuracy and robustness.

The classifier outputs the predicted class y^∈{0,1}, where:

$$\hat{y}=\arg\underset{c\in \{0,1\}}{\max}\sum_{i=1}^{T}I(h_{i}(x)=c)$$

- h*_i_*(x): prediction from the *i*th tree.
- *T*: total number of trees.
- *I*: indicator function (returns 1 if the condition is true)

Hyperparameters:

- Number of trees: 500
- Max depth: optimized using grid search
- Minimum samples per leaf: 5
- Criterion: Gini impurity

Model performance was evaluated on a held-out 20% test set.

#### 2.9.4 Model B – logistic regression for stroke risk

We employed a logistic regression model to estimate the probability of symptomatic stroke:

$$P(y=1|X)=\frac{1}{1+e^{-(\beta_{0}+\beta_{1}X_{1}+\cdot \cdot \cdot +\beta_{n}X_{n})}}$$

Where

- y∈{0,1} is the binary outcome (stroke: yes/no).
- X*_i_* are the predictor variables.
- β*_i_* are regression coefficients estimated via maximum likelihood

Multicollinearity was assessed using the Variance Inflation Factor (VIF); variables with VIF >5 were excluded or regularized.

#### 2.9.5 Model evaluation metrics

Model performance was evaluated using the following metrics:

- Accuracy:
  $$\mathrm{Accuracy}=\frac{TP+TN}{TP+TN+FP+FN}$$
- Precision (Positive Predictive Value):
  $$\mathrm{Precision}=\frac{TP}{TP+FP}$$
- Recall (Sensitivity):
  $$\mathrm{Recall}=\frac{TP}{TP+FN}$$
- **F1 Score**: Harmonic mean of precision and recall
  $$F1=2.\frac{\mathrm{Precision}\cdot \mathrm{Recall}}{\mathrm{Precision}+\mathrm{Recall}}$$
- Area Under the Receiver Operating Characteristic Curve (AUC-ROC): Evaluates discrimination ability across all classification thresholds.

#### 2.9.6 Cross-validation and feature importance

To minimize overfitting, all models were trained using 5-fold cross-validation. For Random Forests, Gini-based feature importance was extracted. For logistic regression, odds ratios and *p*-values were reported.

#### 2.9.7 Machine-learning model development and validation

To explore predictive patterns between atrial fibrillation and CSVD markers, supervised machine-learning models were developed using structured clinical and neuroimaging variables. The dataset was randomly partitioned into training (e.g., 70%) and testing (30%) sets. Model development was conducted exclusively within the training set.

A k-fold cross-validation strategy (e.g., 5-fold or 10-fold cross-validation) was implemented within the training dataset to optimize hyperparameters and evaluate internal model stability. Feature selection procedures were performed within each cross-validation fold to prevent information leakage.

Model performance was evaluated using standard metrics including:

- Area under the receiver operating characteristic curve (AUC)
- Accuracy
- Sensitivity
- Specificity
- Precision and F1-score (where applicable)

Overfitting was minimized through cross-validation, regularization techniques (e.g., L1/L2 penalty where applicable), and strict separation of training and testing datasets. Final model performance was reported on the independent testing dataset to provide an unbiased estimate of predictive accuracy.

#### 2.9.8 Software and implementation

All analyses were conducted using Python (version 3.11) and R (version 4.2.0). Machine learning analyses were implemented in Python using the scikit-learn library (version 1.3), including the *RandomForestClassifier* for model construction, *train_test_split* for data partitioning, and *StandardScaler* for feature normalization. Statistical modeling was performed using statsmodels (version 0.14), specifically logistic regression. R (version 4.2.0) was additionally employed for independent validation and summary statistical analyses.

### 2.10. Ethical consent

This study was conducted in accordance with the Declaration of Helsinki and received ethical approval from the Ethics Committee of our hospital. All participants provided written informed consent prior to inclusion in the study. For participants with cognitive impairment or dementia, informed consent was obtained from a legally authorized representative. Participants were fully informed about the purpose of the study, the procedures involved, potential risks, and their right to withdraw at any point without any impact on their standard medical care. All data were anonymized, securely stored, and handled in compliance with local and international data protection regulations.

## 3 Result

### 3.1 Participant characteristics by brain MRI participation

To clearly distinguish confirmatory hypothesis-driven analyses from exploratory predictive modeling, the Results section is organized into two components. Sections 3.1–3.6 present predefined regression analyses examining associations between AF and CSVD markers. Section 3.7 presents exploratory machine-learning models developed to evaluate predictive performance and feature importance, which should be interpreted as complementary and hypothesis-generating. Among the 1,358 invited individuals, 923 (68%) participated in brain MRI, while 435 (32%) did not (Table 2). A comparative analysis of baseline characteristics between MRI participants and non-participants revealed several statistically significant differences. Gender distribution differed significantly between the two groups. Women were more likely to be non-participants (59.3%) compared to MRI participants (51.9%) (*p* = 0.016), indicating a modest male predominance among those who completed imaging. Educational attainment was higher among MRI participants. A significantly greater proportion of participants (86.6%) had more than mandatory education compared to non-participants (78.6%) (*p* < 0.001), suggesting that higher educational level may be associated with study engagement or health-seeking behavior. Lifestyle factors also varied between groups. Current smoking was less common among participants (7.0%) compared to non-participants (13.3%) (*p* = 0.002), and alcohol risk consumption (>98 g/week) was slightly more frequent in participants (33.0%) than in non-participants (27.4%) (*p* = 0.045). These findings may reflect selection bias favoring healthier or more health-conscious individuals. In terms of clinical parameters, the two groups had comparable systolic and diastolic blood pressure (SBP and DBP), body mass index (BMI), and APOE ε4 allele frequency, with no statistically significant differences (*p* > 0.05). Mean BMI was slightly lower in participants (25.7 ± 4.5 kg/m²) than non-participants (26.5 ± 4.9 kg/m²), though not statistically significant (*p* = 0.120). Cognitive performance, as measured by the Mini-Mental State Examination (MMSE), was notably better among MRI participants (mean: 29.7 ± 1.8) compared to non-participants (28.1 ± 2.9) (*p* = 0.001). This may suggest cognitive status as a key determinant of MRI compliance. Regarding cardiovascular and neurological comorbidities, participants had lower rates of:

- Heart failure (3.7% vs. 6.4%, *p* = 0.045),
- History of symptomatic stroke (5.9% vs. 9.0%, *p* = 0.037), and
- Dementia (1.8% vs. 4.8%, *p* = 0.008),

**Table 2:** Participant characteristics by participation in brain MRI.

Characteristic	Participated in brain MRI (*n* = 923)	Did not participate (*n* = 435)	*p* Value
Female, (%)	479 (51.9%)	258 (59.3%)	0.016
More than mandatory education, (%)	799 (86.6%)	342 (78.6%)	<0.001
Current smoker, (%)	65 (7.0%)	58 (13.3%)	0.002
Alcohol risk consumption >98 g/week, (%)	305 (33.0%)	119 (27.4%)	0.045
BMI, mean ± SD (kg/m²)	25.7 ± 4.5	26.5 ± 4.9	0.120
Systolic BP (SBP), mean ± SD (mm Hg)	138.9 ± 17.9	140.7 ± 21.1	0.287
Diastolic BP (DBP), mean ± SD (mm Hg)	78.7 ± 9.4	80.1 ± 10.8	0.066
MMSE score, mean ± SD	29.7 ± 1.8	28.1 ± 2.9	0.001
APOE ε4 allele, (%)	318 (34.5%)	134 (30.8%)	0.217
Atrial fibrillation, (%)	85 (9.2%)	51 (11.7%)	0.142
Myocardial infarction, (%)	59 (6.4%)	41 (9.4%)	0.081
Heart failure, (%)	34 (3.7%)	28 (6.4%)	0.045
History of symptomatic stroke, (%)	54 (5.9%)	39 (9.0%)	0.037
Dementia, (%)	17 (1.8%)	21 (4.8%)	0.008
Treatment for hypertension, (%)	352 (38.2%)	202 (46.4%)	0.009
Treatment for diabetes, (%)	87 (9.4%)	49 (11.3%)	0.292
Lipid-lowering drug use, (%)	241 (26.1%)	127 (29.2%)	0.213
Anticoagulant treatment, (%)	48 (5.2%)	35 (8.0%)	0.062

suggesting that healthier individuals were more likely to undergo imaging. Although not statistically significant, the prevalence of atrial fibrillation (AF) (9.2% vs. 11.7%, *p* = 0.142) and myocardial infarction (6.4% vs. 9.4%, *p* = 0.081) trended higher among non-participants. In terms of medication use, participants were less likely to receive treatment for hypertension (38.2% vs. 46.4%, *p* = 0.009), whereas use of anticoagulants, lipid-lowering therapy, and diabetes medication did not differ significantly between the two groups (*p* > 0.05). These findings highlight important demographic, cognitive, and clinical differences between individuals who underwent brain MRI and those who did not. Selection bias should be considered when interpreting imaging-based associations in this cohort.

### 3.2 Characteristics of participants with atrial fibrillation

Among the 923 individuals who participated in brain MRI, 85 (9.2%) had a diagnosis of AF (Table 3). Participants with AF demonstrated a distinct clinical and imaging profile compared to those without AF (*n* = 838), reflecting greater vascular and cognitive burden. Sociodemographically, participants with AF were significantly more likely to be male, with only 34.1% being female compared to 53.7% in the non-AF group (*p* = 0.001), suggesting a male predominance in AF prevalence in this cohort. Educational status, however, was similar between groups; 83.5% of AF participants had more than mandatory education vs. 86.9% among non-AF, a non-significant difference (*p* = 0.398). In terms of lifestyle factors, current smoking and alcohol risk consumption (>98 g/week) were numerically higher in the AF group (10.6% and 37.6%, respectively) than in non-AF individuals (6.7% and 32.6%), though these differences did not reach statistical significance (*p* = 0.169 and *p* = 0.358, respectively). Body mass index (BMI) was significantly higher among AF participants (26.9 ± 4.9 kg/m^2^) compared to those without AF (25.6 ± 4.4 kg/m^2^, *p* = 0.041), indicating a higher cardiometabolic burden in this group. Cardiovascular comorbidities were markedly more prevalent among AF participants. Heart disease was present in 30.6% of the AF group compared to just 5.8% in the non-AF group (*p* < 0.001). Similarly, the prevalence of hypertension was nearly double in the AF group (68.2%) vs. the non-AF group (35.1%, *p* < 0.001). Diabetes was also more common among those with AF (22.4% vs. 8.1%, *p* < 0.001), as was hypercholesterolemia (74.1% vs. 55.6%, *p* = 0.003). These findings reflect the well-established clustering of vascular risk factors among individuals with AF. Cognitive impairment was more frequently observed in the AF group. Dementia was present in 4.7% of AF participants compared to only 1.6% in non-AF participants (*p* = 0.041), despite similar distribution of the APOE ε4 allele (35.3% in AF vs. 34.5% in non-AF, *p* = 0.891). The CHA_2_DS_2_-VASc score, a clinical measure of stroke risk, was significantly higher in the AF group (mean 3.3 ± 1.4 vs. 2.2 ± 1.1, *p* < 0.001), aligning with their higher vascular comorbidity burden. Use of anticoagulant treatment was substantially higher in the AF group (56.5%) compared to only 1.3% among non-AF participants (*p* < 0.001), reflecting adherence to clinical guidelines for stroke prevention in AF. Among anticoagulant users in the AF group, warfarin was more commonly used (*n* = 33) than non-vitamin K antagonist oral anticoagulants (NOACs, *n* = 15), although no statistical comparison was made due to small numbers. Neuroimaging findings revealed significantly elevated cerebral small vessel disease burden among AF participants. Symptomatic stroke was reported in 23.5% of individuals with AF, compared to only 4.1% of those without AF (*p* < 0.001). Large infarcts were detected on MRI in 12.9% of the AF group vs. 2.1% in the non-AF group (*p* < 0.001). Similarly, lacunes were found in 17.6% of AF participants compared to 5.0% in those without AF (*p* < 0.001), and silent brain infarcts were present in 15.3% of the AF group vs. 3.5% of non-AF individuals (*p* < 0.001). Although the presence of cerebral microbleeds (CMBs) was higher in the AF group (14.1% vs. 10.7%), this difference did not reach statistical significance (*p* = 0.329). In summary, participants with atrial fibrillation exhibited a higher burden of vascular risk factors, cognitive impairment, and cerebral small vessel disease—including both clinical stroke and silent neuroimaging markers—compared to those without AF. These findings underscore the substantial impact of AF on brain health in older adults.

**Table 3:** Participant characteristics by atrial fibrillation status.

Characteristic	AF (*n* = 85)	No AF (*n* = 838)	*p* Value
Female, (%)	29 (34.1%)	450 (53.7%)	0.001
More than mandatory education, (%)	71 (83.5%)	728 (86.9%)	0.398
Current smoker, (%)	9 (10.6%)	56 (6.7%)	0.169
Alcohol risk consumption, (%)	32 (37.6%)	273 (32.6%)	0.358
BMI, mean ± SD, kg/m²	26.9 ± 4.9	25.6 ± 4.4	0.041
Heart disease, (%)	26 (30.6%)	49 (5.8%)	<0.001
Hypertension, (%)	58 (68.2%)	294 (35.1%)	<0.001
Diabetes, (%)	19 (22.4%)	68 (8.1%)	<0.001
Hypercholesterolemia, (%)	63 (74.1%)	466 (55.6%)	0.003
Dementia, (%)	4 (4.7%)	13 (1.6%)	0.041
APOE ε4 allele, (%)	29 (35.3%)	289 (34.5%)	0.891
CHA_2_DS_2_-VASc score, mean ± SD	3.3 ± 1.4	2.2 ± 1.1	<0.001
Anticoagulant treatment, (%)	48 (56.5%)	11 (1.3%)	<0.001
Warfarin,	33	9	—
NOACs,	15	2	—
Symptomatic stroke, (%)	20 (23.5%)	34 (4.1%)	<0.001
Large infarcts on MRI, (%)	11 (12.9%)	18 (2.1%)	<0.001
Lacunes on MRI, (%)	15 (17.6%)	42 (5.0%)	<0.001
Silent brain infarcts, (%)	13 (15.3%)	29 (3.5%)	<0.001
Cerebral microbleeds (CMBs), (%)	12 (14.1%)	90 (10.7%)	0.329

### 3.3 Association of atrial fibrillation with stroke and cerebral infarcts

AF was significantly associated with an increased risk of clinical and subclinical cerebrovascular lesions (Table 4). In unadjusted analyses, individuals with AF had markedly higher odds of experiencing symptomatic stroke compared to those without AF (OR: 5.3, 95% CI: 2.5–10.9, *p* < 0.001). This association remained robust after adjustment for demographic and vascular risk factors (adjusted OR: 4.2, 95% CI: 2.0–8.8, *p* < 0.001). When stratified by sex, the association was particularly strong among men (adjusted OR: 6.1, 95% CI: 2.3–15.9, *p* < 0.001), while the effect in women, although elevated, did not reach statistical significance (adjusted OR: 2.3, 95% CI: 0.5–9.6, *p* = 0.258), likely due to limited sample size. Similarly, the presence of large infarcts on MRI was significantly associated with AF. In unadjusted models, the odds of large infarcts were more than sevenfold higher in AF participants (OR: 7.4, 95% CI: 2.4–21.8, *p* < 0.001), and this remained statistically significant after full adjustment (adjusted OR: 4.8, 95% CI: 1.4–14.9, *p* = 0.009). In men, AF was associated with a sixfold increased risk of large infarcts (adjusted OR: 6.0, 95% CI: 1.0–33.1, *p* = 0.041), whereas in women the effect was similar in magnitude but not statistically significant (adjusted OR: 4.3, 95% CI: 0.7–25.1, *p* = 0.112). Lacunar infarcts were also significantly associated with AF. The unadjusted OR was 3.6 (95% CI: 1.5–7.4, *p* < 0.001), and the association remained statistically significant after adjustment (adjusted OR: 2.8, 95% CI: 1.1–5.9, *p* = 0.011). Stratified analyses showed that in men, the adjusted odds of lacunes in those with AF were 2.9 times higher than in those without (95% CI: 1.0–8.4, *p* = 0.046). In contrast, no significant association was found in women (adjusted OR: 1.3, 95% CI: 0.2–6.3, *p* = 0.725). A similar pattern was observed for silent brain infarcts. AF was associated with over a fourfold increased odds of silent infarcts in unadjusted models (OR: 4.3, 95% CI: 2.0–8.6, *p* < 0.001), and this association remained significant after multivariable adjustment (adjusted OR: 3.4, 95% CI: 1.4–7.1, *p* = 0.002). Among men, AF was associated with a significantly elevated risk of silent infarcts (adjusted OR: 3.7, 95% CI: 1.3–10.2, *p* = 0.014), whereas in women the association was weaker and not statistically significant (adjusted OR: 2.0, 95% CI: 0.4–8.5, *p* = 0.320). Together, these findings demonstrate that AF is independently associated with both clinically overt and silent cerebrovascular injury, particularly in men. The results suggest a strong and consistent vascular impact of AF on brain structure, with implications for cognitive and functional outcomes in aging populations.

**Table 4:** Atrial fibrillation in relation to symptomatic stroke and MRI findings of large infarcts, lacunes, and silent brain infarcts.

Outcome	Unadjusted OR (95% CI)	*p* Value	Adjusted OR (95% CI)	*p* Value	Men adjusted OR (95% CI)	*p* Value	Women adjusted OR (95% CI)	*p* Value
Symptomatic stroke	5.3 (2.5–10.9)	<0.001	4.2 (2.0–8.8)	<0.001	6.1 (2.3–15.9)	<0.001	2.3 (0.5–9.6)	0.258
Large infarct	7.4 (2.4–21.8)	<0.001	4.8 (1.4–14.9)	0.009	6.0 (1.0–33.1)	0.041	4.3 (0.7–25.1)	0.112
Lacunes	3.6 (1.5–7.4)	<0.001	2.8 (1.1–5.9)	0.011	2.9 (1.0–8.4)	0.046	1.3 (0.2–6.3)	0.725
Silent brain infarcta	4.3 (2.0–8.6)	<0.001	3.4 (1.4–7.1)	0.002	3.7 (1.3–10.2)	0.014	2.0 (0.4–8.5)	0.320

### 3.4 Atrial fibrillation and white matter hyperintensity (WMH) volumes

**Figure 2:**
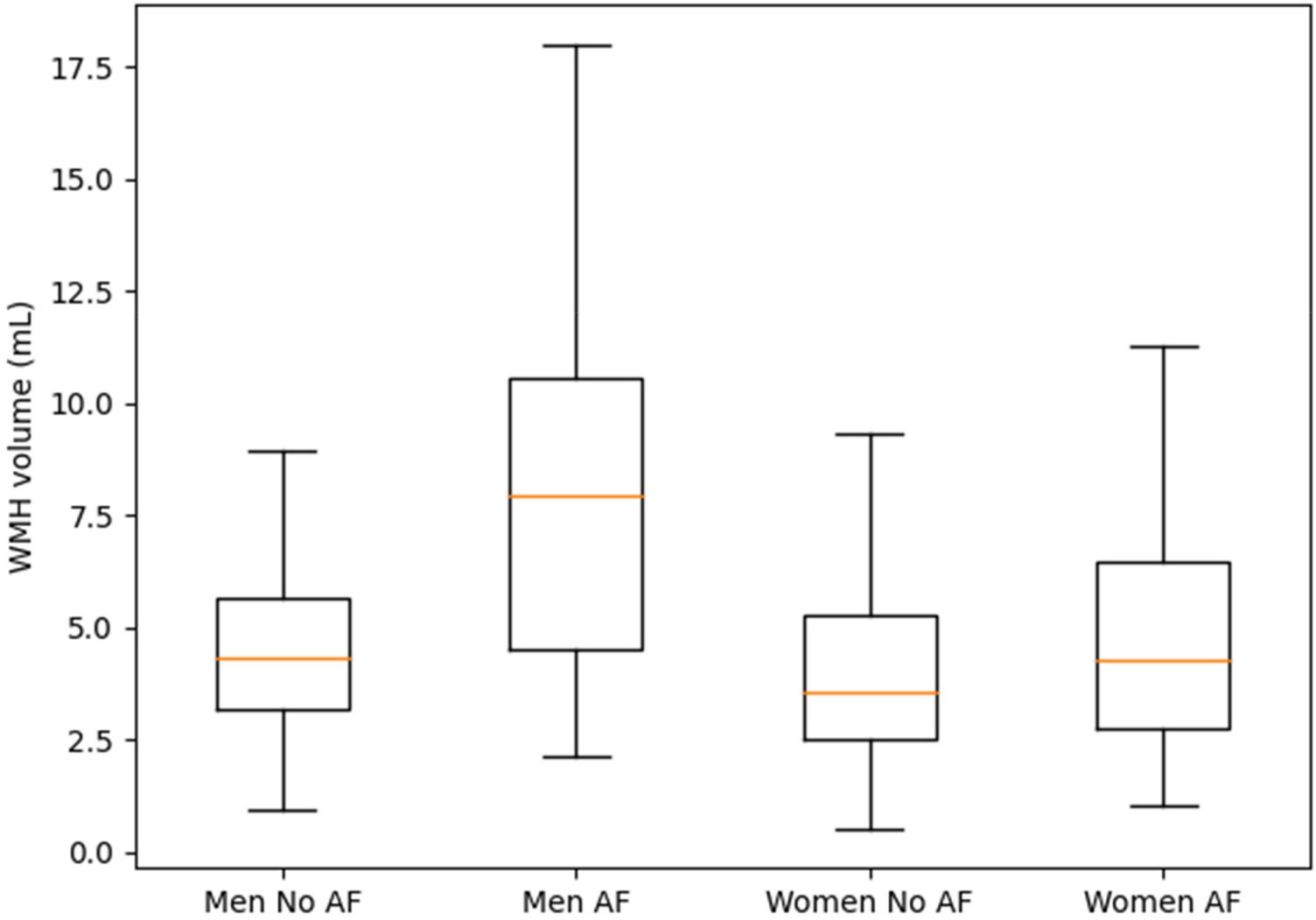
White matter hyperintensity volumes by atrial fibrillation status and sex. Distribution of white matter hyperintensity (WMH) volumes stratified by atrial fibrillation (AF) status and sex. Box plots illustrate median values and interquartile ranges of total WMH volume (mL) among men and women with and without AF. Participants with AF exhibit higher WMH burden compared with those without AF, with a more pronounced difference observed in men.

Participants with AF demonstrated a consistently higher burden of WMHs compared to those without AF (Tables 5, 6). The median uncorrected WMH volume was 5.6 mL (IQR: 2.8–13.9) in the AF group, compared to 4.0 mL (IQR: 2.1–8.0) in those without AF. In the unadjusted model (Model 1), AF was significantly associated with increased WMH volume normalized to total intracranial volume (TIV) (0.0039 mL/TIV vs. 0.0028 mL/TIV, *p* = 0.018). After adjusting for demographic and vascular risk factors (Model 2), the association remained marginally significant (0.0046 vs. 0.0034 mL/TIV, *p* = 0.051), suggesting a potential independent effect of AF on cerebral white matter changes. Further adjustments for history of symptomatic stroke (Model 3) and MRI-confirmed infarcts (Model 4) attenuated the association (Model 4: 0.0055 vs. 0.0047 mL/TIV, *p* = 0.273), indicating that part of the increased WMH burden in AF may be mediated through clinical or silent infarcts. When analyses were stratified by sex, the association between AF and WMH volume was more pronounced among men. Male participants with AF had substantially higher WMH volumes than those without AF (median 7.1 mL vs. 4.5 mL), and this was reflected in the unadjusted model (0.0043 vs. 0.0029 mL/TIV, *p* = 0.029). While the association in men remained elevated in all adjusted models, it did not reach statistical significance (Model 2: 0.0051 vs. 0.0037, *p* = 0.072; Model 4: 0.0061 vs. 0.0048 mL/TIV, *p* = 0.321). In contrast, the association was not significant in women across any model. Female participants with AF had only a slightly higher WMH volume than non-AF women (median 4.2 mL vs. 3.6 mL; Model 2: 0.0037 vs. 0.0030 mL/TIV, *p* = 0.502), and the difference remained nonsignificant after further adjustments (Model 4: 0.0043 vs. 0.0041 mL/TIV, *p* = 0.844). An expanded analysis considering anticoagulation use and age of AF onset (Table 6) revealed important modifiers of WMH burden. Participants with AF who were on anticoagulant therapy had the highest WMH volume among all groups. Their median WMH volume was 8.6 mL, compared to 4.0 mL in non-AF individuals and 3.8 mL in AF participants not on anticoagulants. The adjusted WMH volume in anticoagulated AF participants was significantly elevated (Model 2: 0.0064 vs. 0.0035 mL/TIV in non-AF, *p* = 0.007), and remained so even after adjusting for stroke (Model 3: 0.0071, *p* = 0.021). Although attenuated in the fully adjusted infarct model, the association remained of borderline significance (Model 4: 0.0078, *p* = 0.059). In contrast, AF participants without anticoagulation did not significantly differ from non-AF individuals in any model (Model 2: 0.0033 vs. 0.0035, *p* = 0.675), suggesting that anticoagulation status may reflect either treatment effect or a marker of more advanced vascular disease requiring intervention. Stratification by age at AF onset showed that individuals with early-onset AF (<75 years) had greater WMH volumes (median 6.2 mL) than those with late-onset AF (≥75 years) (median 4.5 mL). In adjusted models, early-onset AF was associated with elevated WMH volumes (Model 2: 0.0050 vs. 0.0041 mL/TIV), although the differences did not reach conventional levels of significance (*p* = 0.071 and *p* = 0.285 in Models 2 and 4, respectively). This trend suggests a possible cumulative effect of long-standing AF on cerebral white matter damage. In summary, atrial fibrillation was associated with increased WMH burden, particularly among men and those on anticoagulant therapy. The relationship was partially mediated by stroke and infarct history, indicating shared vascular pathways. Early-onset AF also tended to show higher WMH volumes, underscoring the potential long-term cerebral impact of chronic arrhythmia. Figure 2 visually demonstrates the higher WMH burden associated with atrial fibrillation, particularly among male participants, supporting the regression results shown in Tables 5, 6.

**Table 5:** AF in relation to WMH volumes.

Group	Uncorrected WMH volumes, median (IQR), mL	Estimated WMH volumes model 1 (Unadjusted), mL/TIV (95% CI)	*p* Value	Model 2 (Adjusted), mL/TIV (95% CI)	*p* Value	Model 3 (Adj. + Stroke), mL/TIV (95% CI)	*p* Value	Model 4 (Adj. + Infarcts), mL/TIV (95% CI)	*p* Value
No AF	4.0 (2.1–8.0)	0.0028 (0.0026–0.0030)	Ref	0.0034 (0.0029–0.0040)	Ref	0.0041 (0.0033–0.0051)	Ref	0.0047 (0.0039–0.0059)	Ref
AF	5.6 (2.8–13.9)	0.0039 (0.0031–0.0052)	0.018	0.0046 (0.0034–0.0061)	0.051	0.0052 (0.0037–0.0070)	0.127	0.0055 (0.0039–0.0075)	0.273
Men No AF	4.5 (2.4–9.2)	0.0029 (0.0027–0.0032)	Ref	0.0037 (0.0030–0.0047)	Ref	0.0043 (0.0034–0.0062)	Ref	0.0048 (0.0039–0.0060)	Ref
Men AF	7.1 (3.5–18.2)	0.0043 (0.0030–0.0062)	0.029	0.0051 (0.0035–0.0075)	0.072	0.0059 (0.0041–0.0085)	0.189	0.0061 (0.0043–0.0088)	0.321
Women No AF	3.6 (1.7–6.9)	0.0025 (0.0022–0.0028)	Ref	0.0030 (0.0025–0.0039)	Ref	0.0036 (0.0028–0.0050)	Ref	0.0041 (0.0032–0.0055)	Ref
Women AF	4.2 (2.1–12.1)	0.0032 (0.0022–0.0048)	0.336	0.0037 (0.0026–0.0054)	0.502	0.0041 (0.0029–0.0061)	0.698	0.0043 (0.0030–0.0064)	0.844

**Table 6:** WMH volumes by AF status, anticoagulation use, and age at AF onset.

Group	Uncorrected WMH volumes, median (IQR), mL	Model 1 (Unadjusted), mL/TIV (95% CI)	*p* Value	Model 2 (Adjusted), mL/TIV (95% CI)	*p* Value	Model 3 (Adj. + Stroke), mL/TIV (95% CI)	*p* Value	Model 4 (Adj. + Infarcts), mL/TIV (95% CI)	*p* Value
No AF	4.0 (2.1–7.8)	0.0029 (0.0026–0.0031)	Ref	0.0035 (0.0029–0.0041)	Ref	0.0041 (0.0033–0.0052)	Ref	0.0046 (0.0037–0.0059)	Ref
AF w/o AC	3.8 (2.0–9.3)	0.0030 (0.0021–0.0044)	0.803	0.0033 (0.0023–0.0049)	0.675	0.0036 (0.0025–0.0054)	0.597	0.0040 (0.0027–0.0057)	0.482
AF with AC	8.6 (4.5–17.8)	0.0052 (0.0036–0.0075)	0.001	0.0064 (0.0044–0.0092)	0.007	0.0071 (0.0049–0.0106)	0.021	0.0078 (0.0053–0.0111)	0.059
AF onset ≥75 y	4.5 (2.3–11.9)	0.0034 (0.0023–0.0050)	0.218	0.0041 (0.0029–0.0061)	0.551	0.0046 (0.0031–0.0069)	0.709	0.0050 (0.0033–0.0074)	0.821
AF onset <75 y	6.2 (3.5–15.6)	0.0041 (0.0030–0.0060)	0.049	0.0050 (0.0036–0.0071)	0.071	0.0056 (0.0040–0.0080)	0.193	0.0060 (0.0042–0.0085)	0.285

### 3.5 Atrial fibrillation and cerebral microbleeds (CMBs)

The association between AF and the presence of CMBs was explored across multiple brain regions using logistic regression models, progressively adjusted for potential confounders including demographic factors, vascular risk profiles, symptomatic stroke, and infarcts (Table 7). In the overall analysis, AF was not significantly associated with the presence of any CMBs. In the unadjusted model (Model 1), the odds ratio (OR) for any CMB in individuals with AF compared to those without was 1.3 (95% CI: 0.6–2.7, *p* = 0.461). This association weakened further with successive adjustments. In the fully adjusted model that included infarct burden (Model 4), the OR decreased to 0.9 (95% CI: 0.4–2.0, *p* = 0.735), suggesting no independent association between AF and the overall presence of microbleeds. Analysis by CMB location similarly revealed no statistically significant associations. For deep CMBs, the unadjusted OR was 1.0 (95% CI: 0.3–3.1, *p* = 0.997), with negligible changes across models (Model 4: OR = 0.7, 95% CI: 0.2–2.4, *p* = 0.643), indicating no link between AF and deep-seated microbleeds. Lobar CMBs, which are typically associated with cerebral amyloid angiopathy and lobar hemorrhagic processes, also showed no significant association with AF. The unadjusted OR was 1.6 (95% CI: 0.7–3.8, *p* = 0.281), which declined in the fully adjusted model (Model 4: OR = 1.0, 95% CI: 0.4–2.6, *p* = 0.980). When the analysis was further stratified into specific lobar regions, a borderline association emerged between AF and frontal lobe CMBs. In the unadjusted model, AF was associated with nearly a threefold increased odds of frontal microbleeds (OR: 2.9, 95% CI: 1.0–8.2, *p* = 0.048). However, this association lost significance after adjusting for confounders. In Model 2, the adjusted OR was 2.5 (95% CI: 0.9–7.4, *p* = 0.078), and it continued to attenuate in Model 4 (OR = 1.9, 95% CI: 0.6–6.3, *p* = 0.288). This suggests that the initial signal may be confounded by other vascular risk factors or cerebrovascular pathology. No significant associations were observed between AF and CMBs in the parietal, temporal, or occipital lobes. The adjusted odds for parietal lobe microbleeds in Model 4 was 1.3 (95% CI: 0.4–4.1, *p* = 0.597), and for temporal lobe microbleeds, 1.1 (95% CI: 0.3–3.5, *p* = 0.831). Occipital lobe CMBs showed a similar lack of association (Model 4: OR = 0.8, 95% CI: 0.2–3.2, *p* = 0.748). In conclusion, atrial fibrillation was not independently associated with the presence of cerebral microbleeds, whether globally or regionally, after controlling for relevant confounders. The initially elevated risk observed in the frontal lobe appeared to be attenuated upon adjustment, indicating that AF may not be a major determinant of microbleed pathology in this population.

**Table 7:** Atrial fibrillation and cerebral microbleeds (CMBs).

CMB location	Model 1 (Unadjusted) OR (95% CI)	*p* Value	Model 2 (Adjusted) OR (95% CI)	*p* Value	Model 3 (Adj. + Stroke) OR (95% CI)	*p* Value	Model 4 (Adj. + Infarcts) OR (95% CI)	*p* Value
Any CMB	1.3 (0.6–2.7)	0.461	1.1 (0.5–2.3)	0.763	1.0 (0.4–2.2)	0.899	0.9 (0.4–2.0)	0.735
Deep CMBs	1.0 (0.3–3.1)	0.997	0.9 (0.2–3.0)	0.862	0.8 (0.2–2.9)	0.771	0.7 (0.2–2.4)	0.643
Lobar CMBs	1.6 (0.7–3.8)	0.281	1.2 (0.5–2.9)	0.697	1.1 (0.4–2.7)	0.815	1.0 (0.4–2.6)	0.980
Frontal Lobe	2.9 (1.0–8.2)	0.048	2.5 (0.9–7.4)	0.078	2.1 (0.7–6.9)	0.172	1.9 (0.6–6.3)	0.288
Parietal Lobe	2.3 (0.8–6.3)	0.114	1.9 (0.6–5.8)	0.277	1.5 (0.5–4.6)	0.445	1.3 (0.4–4.1)	0.597
Temporal Lobe	1.8 (0.6–5.4)	0.283	1.4 (0.5–4.3)	0.523	1.3 (0.4–4.0)	0.672	1.1 (0.3–3.5)	0.831
Occipital Lobe	1.4 (0.4–4.7)	0.615	1.1 (0.3–4.0)	0.885	1.0 (0.3–3.6)	0.974	0.8 (0.2–3.2)	0.748

### 3.6 Atrial fibrillation and cerebral microbleeds in men

Among male participants, the relationship between AF and CMBs exhibited regional variation, with notable findings in specific lobar locations, particularly the frontal and occipital lobes (Table 8). In general, AF was not significantly associated with the presence of any CMBs in men across all models. In the unadjusted model (Model 1), the odds ratio (OR) for any CMB in men with AF compared to those without was 1.6 (95% CI: 0.6–4.0, *p* = 0.328). After full adjustment for confounding variables including stroke and MRI-confirmed infarcts (Model 4), this association declined to an OR of 1.0 (95% CI: 0.4–2.9, *p* = 0.981), indicating no significant overall association between AF and total CMB burden in men. When CMBs were analyzed by location, AF in men was not significantly associated with deep microbleeds, with adjusted ORs remaining nonsignificant across all models (Model 4: OR = 0.9, 95% CI: 0.2–3.9, *p* = 0.923). Similarly, lobar CMBs showed a trend toward increased odds in men with AF in the unadjusted model (OR = 2.4, 95% CI: 0.9–6.3, *p* = 0.089), but this association was weakened and lost statistical significance in adjusted models (Model 4: OR = 1.2, 95% CI: 0.3–4.2, *p* = 0.759). However, regional analysis revealed significant associations in the frontal and occipital lobes. Notably, men with AF had a significantly higher likelihood of having frontal lobe CMBs, even after adjusting for potential confounders. The unadjusted OR for frontal CMBs was 5.8 (95% CI: 1.7–19.2, *p* = 0.005), which remained statistically significant in all adjusted models: Model 2 (OR = 5.0, 95% CI: 1.4–17.5, *p* = 0.014), Model 3 (OR = 4.5, 95% CI: 1.2–16.7, *p* = 0.024), and Model 4 (OR = 3.9, 95% CI: 1.0–15.4, *p* = 0.049). These findings suggest a robust and independent relationship between AF and frontal microbleeds in men. Additionally, a potential association between AF and occipital lobe CMBs was observed in men, though the statistical significance diminished with adjustment. The unadjusted OR was 4.6 (95% CI: 1.2–17.1, *p* = 0.027), suggesting a strong initial link. However, this effect was attenuated in Model 2 (OR = 2.8, *p* = 0.198) and no longer statistically significant in the fully adjusted Model 4 (OR = 2.2, 95% CI: 0.4–11.5, *p* = 0.351). This indicates that the initial association may be confounded by stroke, infarcts, or other underlying vascular factors. The associations between AF and temporal and parietal lobe CMBs followed a similar trend of elevated but statistically non-significant ORs. For instance, temporal lobe CMBs had an unadjusted OR of 2.9 (95% CI: 0.8–10.1, *p* = 0.092), which declined to 1.4 in the fully adjusted model (*p* = 0.644). Similarly, parietal lobe CMBs in men with AF had an unadjusted OR of 3.1 (95% CI: 0.9–10.4, *p* = 0.071), reduced to 1.6 in the fully adjusted model (*p* = 0.451). In conclusion, while AF was not associated with total or deep CMBs in men, a significant and independent association was observed between AF and frontal lobe microbleeds, even after adjusting for stroke and infarcts. Occipital lobe CMBs also appeared more common in men with AF, though this association did not persist after full adjustment. These findings suggest that AF may have a region-specific impact on microvascular brain pathology in men, particularly in anterior lobar regions.

**Table 8:** Atrial fibrillation in relation to CMBs in men.

CMB location (Men)	Model 1 (Unadjusted) OR (95% CI)	*p* Value	Model 2 (Adjusted) OR (95% CI)	*p* Value	Model 3 (Adj. + Stroke) OR (95% CI)	*p* Value	Model 4 (Adj. + Infarcts) OR (95% CI)	*p* Value
Any CMB	1.6 (0.6–4.0)	0.328	1.4 (0.5–3.7)	0.512	1.3 (0.5–3.6)	0.601	1.0 (0.4–2.9)	0.981
Deep CMBs	1.7 (0.5–5.8)	0.379	1.3 (0.4–4.8)	0.667	1.1 (0.3–4.2)	0.845	0.9 (0.2–3.9)	0.923
Lobar CMBs	2.4 (0.9–6.3)	0.089	1.7 (0.6–5.0)	0.310	1.6 (0.5–4.8)	0.382	1.2 (0.3–4.2)	0.759
Temporal CMBs	2.9 (0.8–10.1)	0.092	2.1 (0.6–7.9)	0.264	1.9 (0.5–7.3)	0.367	1.4 (0.3–6.6)	0.644
Parietal CMBs	3.1 (0.9–10.4)	0.071	2.2 (0.6–8.5)	0.245	2.0 (0.5–7.9)	0.318	1.6 (0.4–7.1)	0.451
Frontal CMBs	5.8 (1.7–19.2)	0.005	5.0 (1.4–17.5)	0.014	4.5 (1.2–16.7)	0.024	3.9 (1.0–15.4)	0.049
Occipital CMBs	4.6 (1.2–17.1)	0.027	2.8 (0.6–13.4)	0.198	2.5 (0.5–12.0)	0.277	2.2 (0.4–11.5)	0.351

### 3.7 Exploratory predictive modeling (machine learning analyses)

**Figure 3:**
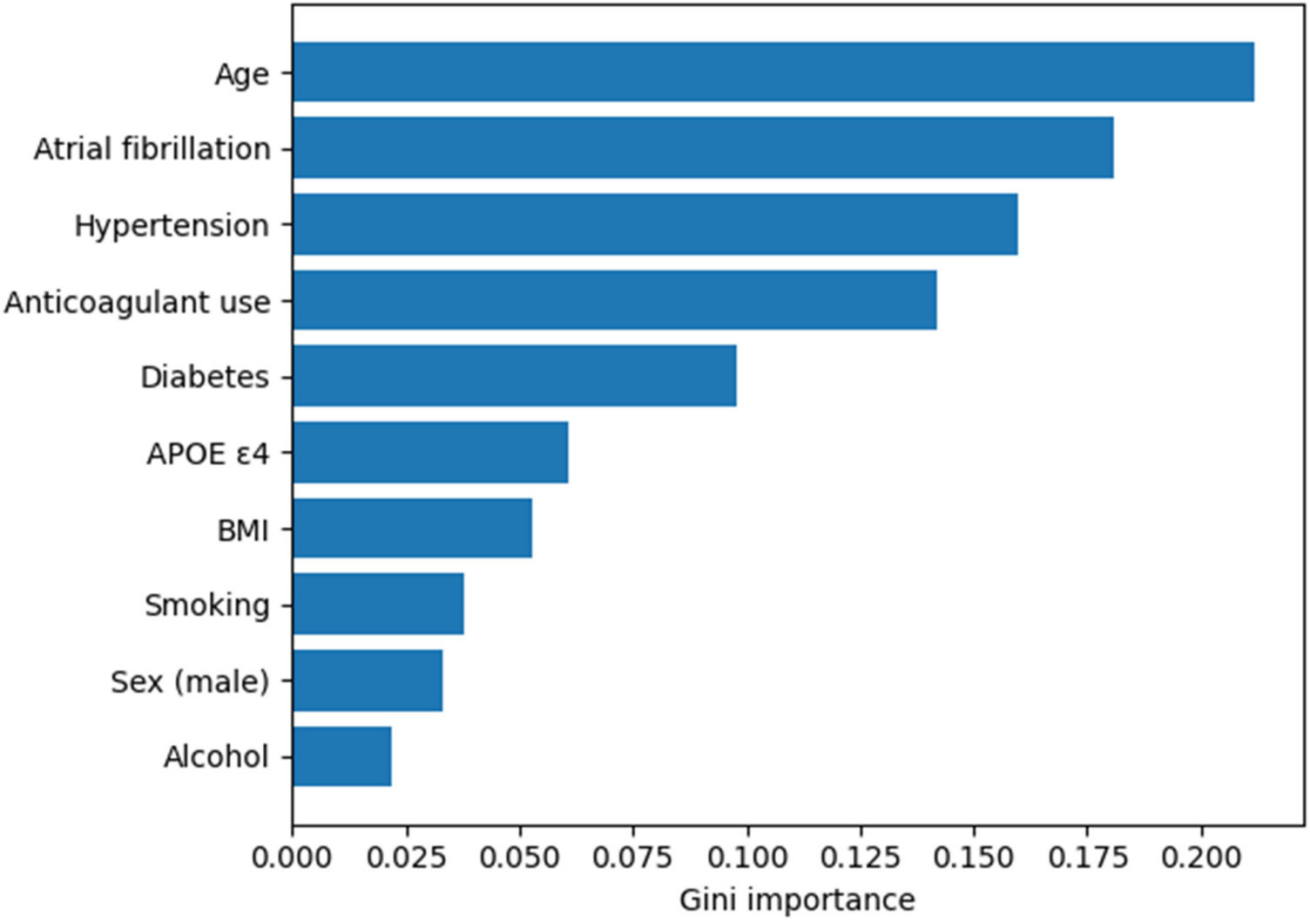
Feature importance from the random forest model predicting high WMH burden. Feature importance derived from the Random Forest model for predicting high white matter hyperintensity (WMH) burden. Variable importance was quantified using Gini impurity reduction. Age, atrial fibrillation, hypertension, and anticoagulant use were the strongest contributors to model performance.

In addition to hypothesis-driven regression analyses, we conducted exploratory machine-learning analyses to assess whether integrated clinical and demographic variables could predict CSVD-related outcomes. These models were not designed to test causal associations but rather to evaluate predictive performance and relative feature importance within this cross-sectional dataset. To explore the potential of clinical and demographic variables in predicting CSVD outcomes, we trained two supervised machine learning models targeting WMH burden and symptomatic stroke risk. The Random Forest model (Model A) was used to classify individuals with high WMH volumes, while logistic regression (Model B) was applied to predict symptomatic stroke occurrence. In Model A, the Random Forest classifier achieved strong performance on the test set, with an overall accuracy of 81.2%, precision of 78.3%, sensitivity (recall) of 83.1%, and an AUC-ROC of 0.88, indicating good discriminative ability (Table 9). The confusion matrix (Table 10) demonstrated balanced classification, with high true positive and true negative rates for WMH prediction. The most influential predictors of high WMH burden, based on Gini importance scores (Table 11), were age (0.212), atrial fibrillation (0.181), hypertension (0.160), and anticoagulant use (0.142). Diabetes, APOE ε4 genotype, BMI, and smoking status contributed modestly, while alcohol use and sex had lower predictive value. Model B, the logistic regression for symptomatic stroke, also performed well, with an accuracy of 84.9%, sensitivity of 68.5%, specificity of 89.1%, and AUC-ROC of 0.91, suggesting excellent model fit (Table 12). The strongest predictor of symptomatic stroke was atrial fibrillation, with an adjusted odds ratio (OR) of 4.2 (*p* < 0.001), reaffirming its independent contribution to cerebrovascular events. Other significant predictors included age (OR = 1.12, *p* = 0.002), hypertension (OR = 2.1, *p* = 0.013), diabetes (OR = 1.8, *p* = 0.021), and male sex (OR = 1.6, *p* = 0.042) (Table 13). Anticoagulant use was associated with a trend toward protective effect (OR = 0.64, *p* = 0.056), while APOE ε4 status, alcohol use, and BMI showed weaker or nonsignificant associations. Together, these models illustrate that integrating atrial fibrillation status with vascular, metabolic, and demographic variables enables accurate prediction of key CSVD outcomes in older adults. The findings align with observed clinical associations in the main cohort analysis and reinforce the relevance of atrial fibrillation, especially in conjunction with hypertension and aging, in the progression of subclinical and clinical cerebrovascular disease. As shown in Figure 3, atrial fibrillation emerged as one of the most influential predictors of high WMH burden, second only to age, reinforcing its central role in cerebral small vessel disease risk.

**Table 9:** Performance metrics for WMH prediction (random forest model A).

Metric	Value
Accuracy	81.2%
Precision	78.3%
Recall (Sensitivity)	83.1%
Specificity	79.0%
F1 Score	80.6%
AUC-ROC	0.88

**Table 10:** Confusion matrix – WMH classification (random forest model A).

Actual Class	Predicted: Low WMH	Predicted: High WMH
Actual: Low WMH	312	41
Actual: High WMH	38	321

**Table 11:** Feature importance for predicting high WMH burden (random forest model A).

Rank	Feature	Importance score
1	Age	0.212
2	Atrial fibrillation	0.181
3	Hypertension	0.160
4	Anticoagulant use	0.142
5	Diabetes	0.098
6	APOE ε4 genotype	0.061
7	Body mass index (BMI)	0.053
8	Smoking	0.038
9	Sex (male)	0.033
10	Alcohol consumption	0.022

**Table 12:** Performance metrics for symptomatic stroke prediction (logistic regression model B).

Metric	Value
Accuracy	84.9%
Precision	76.1%
Recall (Sensitivity)	68.5%
Specificity	89.1%
F1 Score	72.1%
AUC-ROC	0.91

**Table 13:** Odds ratios for predicting symptomatic stroke (logistic regression model B).

Rank	Predictor	Odds ratio (OR)	*p*-value
1	Atrial fibrillation	4.2	<0.001
2	Age	1.12	0.002
3	Hypertension	2.1	0.013
4	Diabetes	1.8	0.021
5	Anticoagulant use	0.64	0.056
6	APOE ε4 genotype	1.3	0.138
7	Smoking	1.2	0.201
8	Body mass index (BMI)	1.05	0.064
9	Sex (male)	1.6	0.042
10	Alcohol consumption	1.1	0.276

## 4 Discussion

In this cross-sectional population-based study, we applied two complementary analytical approaches: (1) hypothesis-driven multivariable regression models to examine predefined associations between atrial fibrillation and CSVD markers, and (2) exploratory machine-learning models to evaluate predictive performance. The regression analyses provide estimates of association adjusted for confounders, whereas the predictive models assess classification accuracy and relative variable importance without implying causality. This distinction is important for interpretation of our findings.

Although atrial fibrillation was not associated with cerebral microbleeds overall, we observed a significant and independent association with frontal lobe CMBs in men. This region-specific finding may reflect the particular vulnerability of frontal brain regions to chronic cerebral hypoperfusion and impaired autoregulation, both of which have been implicated in atrial fibrillation. Irregular cardiac rhythm and reduced cardiac output in AF can lead to fluctuating cerebral blood flow, with frontal regions located in watershed territories supplied by long penetrating arteries being especially susceptible to subtle ischemic injury. In addition, microembolic phenomena related to AF may preferentially affect anterior circulation territories, contributing to focal microvascular damage. Sex-specific differences in vascular structure, endothelial function, and risk factor burden may further modulate this vulnerability, potentially explaining why this association was observed predominantly in men. Nevertheless, given the modest sample size and the borderline level of statistical significance, these findings should be interpreted cautiously and regarded as hypothesis-generating, warranting confirmation in larger longitudinal studies with detailed perfusion imaging.

Importantly, the cross-sectional nature of this study precludes causal inference regarding the relationship between atrial fibrillation and cerebral small vessel disease. Although we observed significant associations between AF and multiple CSVD markers including white matter hyperintensities, symptomatic stroke, large infarcts, lacunes, and silent brain infarcts—the temporal sequence between AF onset and the development of cerebral lesions cannot be determined. Therefore, our findings should be interpreted as evidence of association rather than causation. It remains possible that shared vascular risk factors, subclinical cerebrovascular pathology, or systemic processes contribute to both AF and CSVD simultaneously. Furthermore, reverse causation cannot be excluded, as pre-existing cerebral small vessel disease may influence autonomic regulation or cardiovascular function, potentially increasing AF susceptibility. Although our models were adjusted for a comprehensive set of demographic and vascular confounders including hypertension, diabetes, hypercholesterolemia, APOE ε4 status, prior stroke, and MRI-confirmed infarcts residual confounding from unmeasured factors (such as AF burden, duration, or inflammatory markers) may persist. Accordingly, our results should be considered hypothesis-generating and supportive of a potential pathophysiological link between AF and cerebral microvascular injury, rather than definitive evidence of a causal pathway. Longitudinal cohort studies with repeated neuroimaging assessments and detailed characterization of AF duration and subtype are necessary to clarify temporal relationships and determine whether AF independently accelerates CSVD progression over time.

Our study found that AF is associated with increased WMH volumes, particularly in men and those on anticoagulant therapy, with adjusted WMH volumes of 0.0046 mL/total intracranial volume (TIV) in AF participants compared to 0.0034 mL/TIV in non-AF individuals (*p* = 0.051). The association was more pronounced in men (*p* = 0.072) and attenuated after adjusting for stroke and infarcts, suggesting partial mediation through ischemic pathways. These results are consistent with prior studies reporting a link between AF and WMH burden. Our findings extend these observations to a population-based cohort of older adults, indicating that AF may contribute to WMH even in the absence of clinical stroke, particularly in men. The association between AF and WMH volume approached statistical significance in the fully adjusted model (*p* = 0.051). This borderline result may partly reflect the influence of strong confounders such as hypertension (the top predictor in the Random Forest model; importance score 0.160) and the modest sample size of the AF group (*n* = 85), which can reduce power to detect smaller effect sizes after extensive covariate adjustment.

An intriguing finding of our study was the higher WMH burden observed among participants with atrial fibrillation receiving anticoagulant therapy. This association remained statistically significant after multivariable adjustment (*p* = 0.007), although it was attenuated after accounting for stroke and infarct burden. Importantly, the number of participants treated with non–vitamin K antagonist oral anticoagulants (NOACs) was small (*n* = 15), limiting statistical power to detect differential effects between anticoagulant classes. Therefore, these results should be interpreted with caution. The observed association likely reflects confounding by indication, whereby individuals prescribed anticoagulation represent a subgroup with higher baseline vascular risk, longer AF duration, or greater comorbidity burden, rather than a direct adverse effect of anticoagulant therapy on white matter integrity. Ongoing and recent randomized clinical trials investigating anticoagulation strategies in atrial fibrillation, including comparisons between NOACs and warfarin, may provide important insights into their long-term effects on cerebral small vessel disease and white matter progression. Longitudinal studies with larger samples and repeated neuroimaging will be essential to clarify the causal relationship between anticoagulation and WMH burden.

The stronger association in men aligns with evidence of sex differences in CSVD. Our finding that anticoagulation use was associated with higher WMH volumes (*p* = 0.007) is notable and may reflect either a treatment effect or a marker of more severe vascular disease requiring anticoagulation. However, our data showed no significant difference in WMH burden between warfarin and non-vitamin K antagonist oral anticoagulants (NOACs), possibly due to the small sample size of NOAC users (*n* = 15). Future studies with larger cohorts are needed to clarify the differential effects of anticoagulant types on WMH progression.

The robust association between AF and symptomatic stroke [adjusted odds ratio [OR]: 4.2, 95% confidence interval [CI]: 2.0–8.8, *p* < 0.001] and silent brain infarcts (adjusted OR: 3.4, 95% CI: 1.4–7.1, *p* = 0.002) corroborates the established cardioembolic risk of AF. Our logistic regression model reinforces this with AF as the strongest predictor of stroke (OR: 4.2, *p* < 0.001) (Table 13). Our study further demonstrates that AF is linked to large infarcts (adjusted OR: 4.8, *p* = 0.009) and lacunes (adjusted OR: 2.8, *p* = 0.011), particularly in men, indicating a broader impact on both large-vessel and small-vessel ischemic pathology. The stronger effect in men (e.g., adjusted OR for stroke: 6.1 in men vs. 2.3 in women) may reflect sex-specific differences in thromboembolism risk or cerebral vascular anatomy.

Contrary to WMHs and infarcts, we found no significant overall association between AF and CMBs (adjusted OR: 0.9, 95% CI: 0.4–2.0, *p* = 0.735). However, a region-specific association was observed in men, with AF linked to frontal lobe CMBs (adjusted OR: 3.9, 95% CI: 1.0–15.4, *p* = 0.049). However, Horstmann et al. found no significant association between AF and CMBs, consistent with our overall null finding. The discrepancy may be attributed to differences in study populations or imaging protocols, as our 3.0-Tesla MRI provided high sensitivity for CMB detection. The frontal lobe-specific association in men may reflect regional vulnerabilities to microembolic or inflammatory processes, as frontal regions are particularly susceptible to hypoperfusion in AF. The lack of association with deep CMBs (OR: 0.7, *p* = 0.643) suggests that AF-related microvascular damage may be more cortical than subcortical, potentially linked to cerebral amyloid angiopathy rather than hypertensive microangiopathy. Our predictive models demonstrated high accuracy for WMH burden (Random Forest: 81.2%, AUC-ROC: 0.88) and stroke risk (logistic regression: 84.9%, AUC-ROC: 0.91), with AF, age, and hypertension as leading predictors. The high predictive power of AF in both models underscores its utility as a clinical marker for CSVD risk stratification. The protective trend of anticoagulant use in stroke prediction (OR: 0.64, *p* = 0.056) is consistent. However, the association of anticoagulation with higher WMH volumes suggests a complex interplay, potentially reflecting underlying disease severity rather than a direct causal effect.

Several limitations should be considered when interpreting our findings. First, the cross-sectional design precludes determination of temporal sequence or causality. Although atrial fibrillation (AF) was associated with multiple CSVD markers, including white matter hyperintensities, infarcts, and symptomatic stroke, we cannot establish whether AF preceded the development of cerebral pathology or whether shared vascular mechanisms contributed to both conditions. Reverse causation is also possible. Longitudinal studies with repeated neuroimaging are necessary to clarify progression and directionality. Second, residual confounding may persist despite comprehensive adjustment. Although models accounted for demographic factors, vascular risk factors, APOE ε4 status, stroke history, and infarcts, unmeasured factors such as AF burden (paroxysmal vs. persistent), AF duration, left atrial size, cardiac output variability, inflammatory markers, socioeconomic status, medication adherence, and blood pressure variability were not available. These factors could influence both AF severity and cerebral microvascular injury. Third, confounding by indication may have influenced associations related to anticoagulation therapy. Participants receiving anticoagulants likely represent a subgroup with higher vascular risk or longer AF duration. Therefore, the observed association between anticoagulation use and higher WMH burden should not be interpreted as evidence of treatment harm but rather as potentially reflecting underlying disease severity. Fourth, selection bias may affect generalizability. Individuals who underwent MRI had higher educational attainment and better cognitive function than non-participants, suggesting a healthier volunteer effect. Additionally, the cohort consisted of adults aged ≥70 years from a specific geographic region in China, which may limit extrapolation to younger populations, other ethnic groups, or healthcare systems with different cardiovascular risk profiles. Fifth, statistical power for certain subgroup analyses was limited. The number of participants with AF (*n* = 85), particularly women with AF and NOAC users, was modest. Consequently, some sex-stratified or region-specific findings especially those with borderline statistical significance should be interpreted cautiously and regarded as hypothesis-generating. Finally, although predictive modeling demonstrated strong discrimination (AUC-ROC up to 0.91), these models were internally validated only. External validation in independent cohorts is necessary before clinical implementation. Model performance and calibration must be confirmed in independent populations with varying demographic, clinical, and imaging characteristics before clinical implementation. Furthermore, correlated neuroimaging features may have introduced residual model instability despite cross-validation safeguards. Overall, while the standardized imaging protocol and well-characterized cohort strengthen internal validity, external validation and longitudinal confirmation are necessary to establish robustness, generalizability, and clinical relevance. Future research should prioritize longitudinal designs to explore the cumulative effects of AF on CSVD, particularly in women, and investigate the role of AF duration and type. Advanced imaging techniques, such as diffusion tensor imaging, could further elucidate microstructural changes associated with AF. Moreover, randomized trials comparing anticoagulation strategies (e.g., NOACs vs. warfarin) could clarify their impact on CSVD progression, addressing the potential trade-off between stroke prevention and microvascular damage.

## 5 Conclusion

This study demonstrates that atrial fibrillation (AF) is independently associated with an increased burden of cerebral small vessel disease (CSVD) markers, including white matter hyperintensities (WMHs), symptomatic stroke, large infarcts, lacunes, and silent brain infarcts, particularly in men. The elevated WMH volumes observed in AF participants, especially those on anticoagulant therapy and with early-onset AF, suggest a cumulative vascular impact of chronic arrhythmia, partially mediated by stroke and infarct pathways. While AF was not significantly associated with cerebral microbleeds (CMBs) overall, a notable region-specific association with frontal lobe CMBs in men highlights potential sex-specific and anatomical vulnerabilities in AF-related microvascular brain injury. These findings underscore the importance of AF as a modifiable risk factor for both clinical and subclinical cerebrovascular damage in older adults. Future research should focus on longitudinal studies to clarify the causal mechanisms linking AF to CSVD and explore targeted interventions, such as optimized anticoagulation strategies and vascular risk factor management, to mitigate AF-related brain pathology and its implications for cognitive and functional decline. Sex-stratified approaches may further enhance personalized prevention and treatment strategies for AF patients at risk of CSVD. However, given the cross-sectional design and the demographic characteristics of the cohort, these findings should be interpreted cautiously and validated in longitudinal and multi-ethnic populations before broader generalization.

## Data Availability

The original contributions presented in the study are included in the article/Supplementary Material, further inquiries can be directed to the corresponding author.
